# A Review of Vitamin A Supplementation in South Sudan: Successes, Challenges, and Opportunities for the Way Forward

**DOI:** 10.9745/GHSP-D-21-00660

**Published:** 2022-06-29

**Authors:** Nancy Jean Haselow, Vandana Joshi, Priscilla Nicholas Bayo, Jesca W. Murye, Sadick Nawal Shaban, Kiross Tefera Abebe, Ismail Kassim, Tesfatsion Shiweredo, Hari Vinathan, Chandrakala Pramod Jaiswal, Khamisa Ayoub Miluwa, Eric Alain Ategbo, Biram Ndiaye, Mohamed Ag Ayoya

**Affiliations:** a UNICEF South Sudan, Neenah, WI, USA.; b UNICEF South Sudan, Juba, The Republic of South Sudan.; c UNICEF Nigeria, Maiduguri, Nigeria.; d UNICEF, Yemen, Sana'a Yemen.; e UNICEF Lao People's Democratic Republic, Vientiane, Lao People's Democratic Republic.; f South Sudan Ministry of Health, Juba, The Republic of South Sudan.; g UNICEF Somalia, Mogadishu, Somalia.; h UNICEF Afghanistan, Kabul, Afghanistan.

## Abstract

**Aim:**

To identify vitamin A supplementation (VAS) trends in South Sudan and provide insights to refocus VAS programming vis a vis polio eradication campaigns recently phased out while access to health care, land, food, and markets remain challenging.

**Method:**

Review of data from survey and coverage reports; review of policy and program documents; key informant responses; general literature search.

**Results:**

Vitamin A deficiency (VAD) is likely a severe public health problem among preschool-aged children in South Sudan based on a high under-5 mortality rate (96.2 deaths/1,000 live births) and high levels of undernutrition, infections, and food insecurity. Vitamin A capsules, with deworming tablets (VASD), have been delivered to preschool-aged children during national immunization days (NIDs) for the past decade. Although areas of South Sudan and certain populations continue to have low VAS coverage, when comparing national VAS coverage (reported in the last 6 months) between 2010 and August 2019, a large improvement is noted from 4% to 76%. In 2021, VAS coverage was more than 90% at the national level during 2 stand-alone distribution campaigns. Deworming coverage trends generally mimicked VAS coverage. VAS is provided to postpartum mothers who deliver at health facilities (approximately 12%–25%), but coverage data are not available.

**Conclusion:**

Twice-yearly VAS should remain a key lifesaving intervention to address VAD, but alternative delivery strategies will be needed. Conducting events, such as child health days, supported by promotional activities or community-based VASD distribution activities for the youngest children and those missed during campaigns, should be considered. For the long term, a hybrid approach targeting underserved areas with mass distribution events while integrating VASD into community-based programs such as quarterly screening for wasting should be tested further and gradually scaled up everywhere as this has the potential to sustainably reach all vulnerable children twice yearly.

## Abstract (precis)

Although South Sudan's vitamin A supplementation program has demonstrated success, vitamin A supplementation remains a critical public health need for young children. How can South Sudan best maintain high vitamin A supplementation coverage for the short to medium term while planning a more sustainable delivery approach for the longer term?

## Teaser

**Key Findings:**

- Undernutrition and high under-5 mortality persist in South Sudan, with very low dietary intake of vitamin A and a high rate of infection considered to be the primary causes of vitamin A deficiency among young children.
- South Sudan's vitamin A supplementation (VAS) program has demonstrated success through campaign-style delivery with steady improvement from 2010 to the present.
- Continued political unrest, environmental factors, and health system infrastructure deficiencies contribute to low and uneven VAS coverage rates at different times and in different areas.

**Key Implications:**

- Program managers should consider developing a flexible, multifaceted delivery mechanism to ensure vitamin A supplementation and deworming (VASD) reach all children everywhere every 6 months.
- To support program managers in decision making, researchers should consider analyzing and using the Food Security and Nutrition Monitoring System data more fully to better target gaps in VASD coverage.
- Policy makers should consider how integrating VASD into community-based programming could strengthen the health system by extending it more fully into boma (villages) and take steps to build VASD into planning and budget cycles at all levels.

## INTRODUCTION

Vitamin A deficiency (VAD) especially affects young children, among whom deficiency can result in clinical problems (xerophthalmia and hearing loss from severe otitis media),^1^ limit growth, weaken innate and acquired host defenses, exacerbate infections, and increase the risk of death.^2^ After the 1990 United Nations' call to virtually eliminate VAD, vitamin A (VA) interventions (primarily vitamin A supplementation [VAS], but also food fortification and dietary diversification) were intensified, contributing to the global reduction of VAD among preschool-aged children from 39% in 1991 to 29% in 2013. In sub-Saharan Africa, however, VAD prevalence has changed little over the past 30 years and remains high at 48%.^3^

It is estimated that 157,000 deaths or 2%–3% of all deaths among children aged younger than 5 years globally are attributable to VAD each year.^4^ Several meta-analyses of randomized controlled efficacy trials showed that providing high-dose vitamin A capsules (VAC) to children aged 6–59 months living in VAD populations increased their chance of survival by reducing all-cause mortality by 23%–24%, measles morbidity and mortality by 50%, diarrheal disease mortality by 28%–33%, and incidence of diarrhea by 15%.^5^^,^^6^ Even with the addition of a large program effectiveness study in India (which was not a randomized controlled trial), the all-cause mortality reduction is still 12%.^7^

Until relatively recently, low- and middle-income countries (LMICs) have addressed VAD primarily by distributing a high-dose VAC every 6 months to children aged 6–59 months and 1 dose to women postpartum (although the latter is no longer recommended by the World Health Organization [WHO]).^8^ VAS has the advantage of supplying a large amount of bioavailable VA at one point in time and is considered one of the most cost-effective health interventions for reducing young child mortality.^9^ However, providing high-dose VAS does not sustain a rise in serum retinol for more than 2–3 months,^10^^,^^11^ and thus should be viewed only as a critical intervention to reduce diarrheal and measles morbidity and save young children's lives in areas with high under-5 mortality rates (U5MR) where VAD is a public health problem, rather than a sole strategy to control VAD.^7^^,^^12^ Although bringing these interventions to scale has proven difficult in LMICs,^13^ maintaining adequate VA intake through dietary diversification, optimal breastfeeding, food fortification, and/or low-dose VAS has been shown to sustain serum retinol levels to normal and must be central in the effort to eliminate VAD as a public health problem in all countries.^10^^,^^11^

Nevertheless, to achieve high VAS coverage in countries with a moderate to high VAD burden and high U5MR, the focus has been to use special events or mass campaigns, such as child health days and/or national immunization days (NIDs), to deliver VAC twice yearly. An analysis of UNICEF's 82 VAD priority country database showed that annual global coverage with 2 doses of VAC steadily increased between 2000 and 2009 (27%–78%) but then dropped in 2016 to 64%, indicating that commitment to and funding for VAS programs was declining. The decrease was concentrated in populations exhibiting high child mortality rates.^14^^,^^15^ Between 2017 and 2019, VAS coverage slightly declined further to 61% but in 2020 fell to 42% among all priority countries and was only 24% among countries with U5MR>60%.^16^

In addition to the coronavirus disease (COVID-19) pandemic, reasons for low coverage include social and political unrest that has limited access in some countries and the scale back of polio immunization campaigns on which VAS had been piggybacked.^14^ Several factors have led some countries to change delivery strategies or end their VAS programs including the perception that VA fortified foods are addressing the problem and a renewed call to strengthen primary health care for sustained delivery of critical health and nutrition services.^17^ Justifiably or not, the campaign approach is viewed as circumventing the health system rather than strengthening it. Unfortunately, VAS coverage when delivered through routine health system contacts has been much lower than through special events, with only 23% of semesters having more than 80% VAS coverage globally and only 10% of semesters having more than 80% VAS coverage in the Eastern and Southern Africa region between 2011 and 2016.^14^ As health systems strengthen, VAS coverage could naturally improve.

A Global Alliance for Vitamin A analysis indicated that 17/82 countries still have at least a moderate VAD public health problem among children aged 6–59 months. South Sudan is one of these countries. Until the health and nutrition situation improves sustainably, mass distribution of high-dose VAC twice yearly is recommended as a critical child survival intervention.^15^

South Sudan faces many challenges toward achieving the recommended high VAS coverage (more than 80%), as well as uptake of other optimal nutrition and health practices. Financial, political, and policy opportunities, however, do exist. A peace agreement signed in 2005 between Sudan and the Sudan People's Liberation Movement formally ended that conflict and created the new nation of the Republic of South Sudan in 2011.^18^ However, internal subnational conflicts in South Sudan have persisted with resultant insecurity, displacement of the population, low food production, high food prices, high unemployment, low literacy, disrepair of infrastructure, disruptions to markets, high inflation rates, and steep currency devaluations. Restricted access to insecure areas and periodic severe flooding (and now the COVID-19 pandemic) have disrupted the delivery of humanitarian assistance and access to health services making it difficult to achieve optimal coverage of lifesaving interventions including VAS for young children.^19^ With the consolidation of the peace process through the formation of a national unity government in February 2020, there is optimism for improving the country's structural, economic, and social development, which will enable vulnerable families to reestablish their livelihoods and better meet their food, nutrition, and health needs.

South Sudan faces many challenges toward achieving the recommended high VAS coverage of more than 80%.

## AIM AND METHODOLOGY

We aim to identify VAS trends in South Sudan and provide insights to refocus VAS programming given that polio eradication campaigns were phased out in 2020 and access to health care, land, food, and markets remains challenging. The methodology, type of data used, and guiding principle for the structure and content of this article are based on the authors' understanding of all elements that impact the delivery of VAS in LMICs. Secondary data from survey reports and coverage reports were the basis as they provided a glimpse of the VAS situation over time. No formal analysis of the qualitative information collected was done. However, program documents outlined the government's public health goals for the country. Key informant questions were used to clarify inconsistencies, fill in knowledge gaps, and add more nuanced information regarding the obstacles faced and how challenges were overcome. The analysis represents the authors' synthesis of inputs from the following sources.

The primary sources of survey data and coverage reports used for this review included the South Sudan Household Health Survey (SHHS) 2006 (representative at the state level);^20^ SHHS II 2010 (representative at the national level);^21^ NID summary coverage reports from tally sheet data (2014–2020) (provided by the South Sudan Ministry of Health [MOH]), Food Security and Nutrition Monitoring System (FSNMS) 2015–2020 survey bulletins, reports, and raw data from FSNMS survey round 26 (provided by UNICEF); and summary coverage tables for 2 VAS and deworming (VASD) stand-alone campaigns in 2021 (provided by UNICEF based on county-level distribution reports from State MOH). FSNMS was designed to collect data twice yearly on children aged younger than 5 years and their mothers, representative (with 95% confidence intervals and a margin of error of 10%) at the national, state, and county levels, and was useful for identifying high- and low-performing areas, assessing trends, and looking for associations between food, nutrition, health, and care indicators.^22^^–^^30^ The 2019 State of Food Insecurity and State of the World's Children provided U5MR and infant mortality rate data.^31^^,^^32^

Key policy and program documents described South Sudan's nutrition context and vision for the future and included the following internal MOH documents: The Basic Package of Health and Nutrition Services in Primary Health Care, the National Guidelines on Integration of Vitamin A Supplementation and Deworming into Routine Screening for Acute Malnutrition, the Maternal, Infant and Young Child Nutrition (MIYCN) Strategy (2017–2025) and MIYCN Guidelines, and the National Health Policy 2016–2026 (Box).

BOX **South Sudan's Nutrition Primary Health Sector Strategy**

The National Health Policy 2016–2026 lays out a strategy to move South Sudan from an emergency health system to sustainable health system development, anchored in equitable access to quality care in all communities.

The MIYCN Strategy 2017–2025 highlights nutrition within the government public health policy framework.

The Basic Package of Health and Nutrition Services in Primary Health Care operationalizes the government's strategic plans and covers preventive, curative, health promotion, and managerial activities for health and nutrition.

Within a decentralized health structure, the intention is that:

State Ministry of Health departments provide leadership, oversight, funding, and overall management (including ensuring supplies).

County health departments (the lowest administrative unit) manage the delivery of primary and secondary health services in their catchment area.

At the secondary and primary levels, care is to be delivered at the following locations:

- County hospitals, serving 150,000–300,000 people
- Primary health care centers (PHCCs) at the county level, serving 50,000 people
- PHCCs at the payam level, serving 25,000 people
- Primary health care units at the boma level, serving 15,000 people

Vitamin A supplementation and deworming (VASD) and a therapeutic nutrition program are part of outreach services by nurses from PHCCs. Community health workers (CHWs) from primary health care units and community nutrition volunteers (CNVs) are trained to give VASD, do social mobilization activities, and organize the village; to provide counseling, education, and promotion of maternal, infant, and young child nutrition; to screen pregnant and lactating women and young children regularly for malnutrition and refer cases for treatment to the various health facilities or designated nutrition sites; and to diagnose and treat selected health problems (e.g., malaria, diarrhea, and acute respiratory infections) and promote family planning, vaccination, mosquito net use, and proper WASH practices as well as record receipt of services.

At the boma level, CNVs and mother-to-mother support group members work with CHWs to provide care in close collaboration with boma health committees. Mother-to-mother support group members are trained to support CHWs and CNVs to conduct all nutrition-related activities. Currently, there are more than 8,000 CNVs and about 4,550 mother-to-mother support group members engaged throughout the country. The goal is to have 1 CNV serving 150 households and 1 mother's support group (of 15 members) for every 2,000 people.

Health services at the primary and secondary levels are meant to be free and eventually accessible to everyone. Unfortunately, funding and continued insecurity are serious constraints to realizing the national health policy and equitable rollout and full implementation of the Basic Package of Health and Nutrition Services. Development partners currently provide considerable financial, technical, and programmatic support and will continue to play an important role in providing critical health and nutrition services, particularly in vulnerable priority areas.

Articles and documents from a general literature search (via PubMed, Google, mining references from relevant articles, and recommendations from experts) provided insight into the continued importance of VAS and the delivery mechanism (campaigns versus integration into the routine health system) as well as describing the South Sudan conflict and its effects on access to health care, food, and economic and social opportunities.

Key informant responses to targeted questions were qualitative in nature and enabled more in-depth understanding of the local context, gleaned insights as to why VAS delivery has been effective or not, filled in context and knowledge gaps, and verified information. Responses came primarily from UNICEF South Sudan staff with input and clarifications by the South Sudan MOH staff, Nutrition Cluster Coordinating Team members, and the FSNMS Technical Team.

**FIGURE 1:**
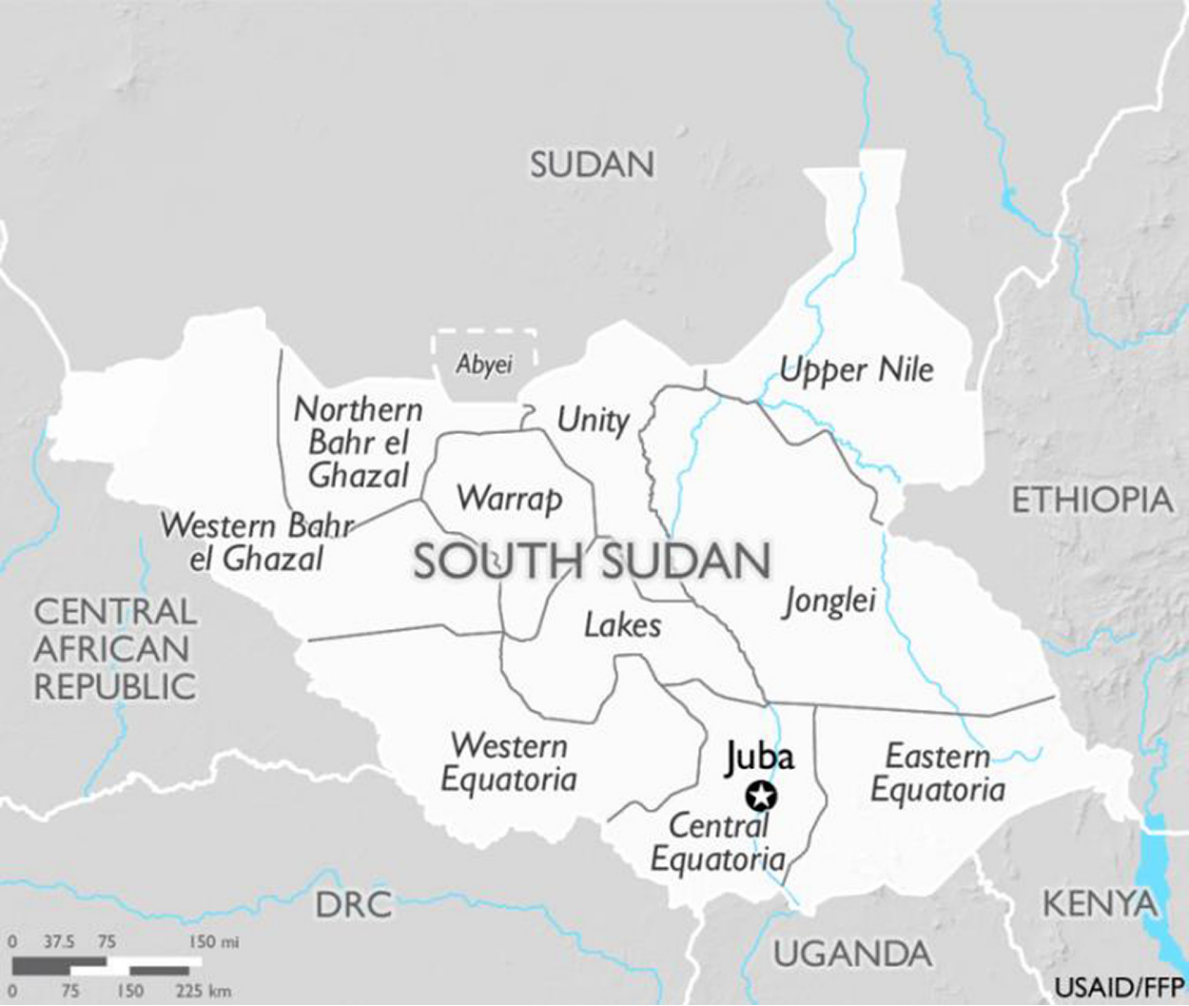
Map of Republic of South Sudan Showing Current Member States

While this analysis is based on considerable available information, it is limited by: inaccessibility to raw data from the FSNMS survey reports that would have allowed for a pooled analysis providing a more in-depth look at trends and associations; by gaps and inconsistencies in data found in reports (although VAS data were collected during each FSNMS Round, VAS coverage was not reported in all survey reports, there were inconsistencies between statistics and narrative in some reports and inconsistencies between the number of children receiving VAC and number of VAC used in some summary tables); and by COVID-19 travel restrictions, limiting in-depth qualitative discussions and field visits to follow the data in-country. Also, note that the population within states and counties has fluctuated considerably and the number of states and counties also changed slightly several times since 2010, but the current 10 states are shown in Figure 1, and these will be referenced throughout the document.

## RESULTS OF THE REVIEW

### VAD in South Sudan

#### Evidence and Classification

There has been no xerophthalmia prevalence or biochemical survey to classify the risk of VAD in South Sudan. However, WHO did a regression analysis for 1995–2005 that predicted a severe VAD problem (<0.70 µmol/L serum retinol among 20% or more of the population) in what was the former Sudan with an estimated 27.8% low serum retinol among children aged 6–59 months and a moderate VAD problem (16.7%) among pregnant women.^33^ Given that the health and nutrition indicators in the southern states were worse,^20^ VAD prevalence would likely have been worse as well.

Undernutrition continues to be a major health problem in South Sudan. The most recent national-level data show that 15.1% of children aged younger than 5 years are stunted with 3.9% severely stunted, and wasting and severe wasting are estimated at 15.6% and 3.5%, respectively, during July/August 2019 (pre-harvest lean season), and at 12.6% and 3.3%, respectively, in December 2019 (post-harvest)—classified as critical to serious in both periods. Women's nutrition status is also poor with 38.2% of women of childbearing age (excluding pregnant women) being underweight (body mass index less than 18.5%) at the national level in December 2019.^30^ In addition, data analysis suggests that high U5MR can serve as a proxy indicator for VAD. Global Alliance for Vitamin A recommends that U5MR of >40 deaths/1,000 live births may guide countries, along with VA dietary intake and infection data, as to when to consider scaling back, targeting, or ending their prophylactic VAS program.^15^ The U5MR in South Sudan has plateaued since 2013 and remains high at 96.2 deaths/1000 live births with boys having a higher U5MR than girls (101 and 91 deaths/1,000 live births, respectively).^34^ Given there is no recent biochemical or xerophthalmia prevalence data to classify risk, the VAD problem in South Sudan among children aged younger than 5 years is currently considered to be at least moderate, but very likely severe, based on the high U5MR, the WHO regression analysis findings, and high rates of undernutrition.

National data show that 15.1% of children aged younger than 5 years are stunted with 3.9% severely stunted, and wasting/severe wasting are estimated at 15.6%/3.5%, respectively.

#### Determinants

Although the cause of VAD can be complex, low dietary intake of VA exacerbated by infection is considered to be the major cause.^35^^,^^36^ Measles, diarrhea, and intestinal parasites, in particular, reduce VA absorption or deplete the already low VA stores that young children have in their bodies.^37^ In 2019, nationally, 54.6% of children aged 0–59 months were reported having 1 or more illnesses (mainly fever, diarrhea, and cough) in the past 2 weeks during the post-harvest period whereas 37.7% reported illness during the pre-harvest lean season.^29^^,^^30^ Estimates of national measles vaccination coverage were low at about 52% annually from 2016 to 2020.^38^ There are little data on intestinal parasites in South Sudan; however, a study showed a prevalence of 18.6% for *Guardia intestinalis* (protozoa) and *Hymenolepis nana* (helminth) among primary and secondary students.^39^ Suboptimal water, sanitation, and hygiene access and practices cause repeated infections (particularly diarrheal episodes and intestinal parasites) exacerbating the fragile health and nutritional status of young children by introducing pathogens repeatedly into their system.^40^ In 2019, at the national level, 34% of families had access to clean water, 23% of the population had access to latrines although only 19% used them regularly, 21% of households had soap to wash hands after defecating or before cooking, and 47% of mothers practiced bottle feeding their children aged 0–23 months.^30^

As in other LMICs, the primary contributor to the ongoing VAD problem in South Sudan is inadequate intake of VA due to high food insecurity and very low dietary diversity that has persisted for over a decade.^41^ The most food insecure time of the year is the pre-harvest period (April–September). However, based on the Consolidated Approach to Reporting Indicators of Food Security, even at harvest time in December 2019, 69.2% of households were food insecure of which 22.6% were severely food insecure. Dietary diversity scores (using the Household Dietary Diversity Score) are also alarming with 38.3% of households at the national level consuming only 0–2 food groups in the past week. At the national level, weekly household consumption of cereals was adequate, but consumption of protein-rich food, dairy, vegetables, and fruits were inadequate (Table 1). Consumption of VA-rich foods is generally low during all seasons everywhere; and oil, needed for absorption of VA, is very low across the country.^29^^,^^30^

**TABLE 1.** Average Days/Week of Household Consumption by Different Food Commodities in South Sudan, December 2019^a^

State	Cereals/Tubers	Pulses	Milk/Dairy	Meat/Fish/Eggs	Vegetable	Fruits	Oil	Sugar	Condiment
Central Equatoria	4.65	1.74	0.26	0.37	3.39	1.50	1.35	0.97	2.75
Eastern Equatoria	6.06	1.18	1.90	0.86	3.68	0.87	2.56	0.87	4.16
Jonglei	4.74	0.76	1.55	0.87	1.18	0.58	1.74	0.74	0.95
Lakes	4.40	2.38	1.09	0.59	2.31	0.25	1.04	1.95	2.19
Northern Bahr el Ghazal	5.77	1.84	1.28	0.96	1.70	0.40	0.32	2.09	3.97
Unity	5.93	0.93	3.56	1.02	0.80	0.19	2.48	1.21	1.55
Upper Nile	4.73	0.73	1.60	1.58	1.19	0.42	1.99	1.57	1.61
Warrap	6.06	2.20	2.17	0.84	1.72	0.60	0.49	0.93	3.29
Western Bahr el Ghazal	5.56	3.16	0.39	0.55	2.41	0.30	0.51	2.71	3.64
Western Equatoria	5.61	2.54	0.21	0.75	3.39	1.85	2.48	1.38	3.49
National	5.29	1.59	1.49	0.85	2.09	0.71	1.54	1.29	2.56

a Data source: Food Security and Nutrition Monitoring System Round 25 Survey Report.

Persistent high food insecurity and very low dietary diversity is the primary contributor to VAD in South Sudan.

Inequitable intrahousehold food distribution and/or suboptimal infant and young child feeding practices appear to limit VA intake among the most vulnerable groups with only 7% of children aged younger than 5 years consuming an adequate diet,^42^ only 4.1% of children aged 6–23 months receiving a minimum acceptable diet, and only 29.7% of women of childbearing age having minimum dietary diversity at the national level.^30^ Although breastfeeding is commonly practiced, breastmilk retinol may be low given that maternal VA dietary intake (which is low) is an important determinant of breastmilk VA concentration.^43^ Although 77% of mothers initiate breastfeeding within 1 hour of birth, many women in rural areas commonly discard colostrum (very rich in VA).^44^ Exclusive breastfeeding to 6 months (early breastmilk is high in VA) is practiced by 68.1% of mothers and prolonged breastfeeding through 1 year is practically universal (92.5%) whereas by age 2 only 58.9% of mothers are still breastfeeding.^30^

### VAS in South Sudan

#### MOH Recommendations

The South Sudan MOH recommends VAS (as a prophylactic dose) for all children aged 6–11 months (100,000 IU) and 12–59 months (200,000 IU) every 6 months.^21^ Considering the many challenges in South Sudan over the past decade, the most effective way to reach children has been through mass campaign-style events and through integrated rapid response mechanisms in areas that are not easily accessible by the government or supporting partners. VAS along with deworming (for children starting at age 12 months) was integrated into polio NIDs from 2010 through 2019 and was piggybacked onto the measles vaccination follow-up campaign in 2020. Since South Sudan was certified as “polio free” in 2020, 2 VASD stand-alone campaigns took place throughout the country in 2021. The campaign approach has enabled a greater number of children to be reached in an emergency environment.^23^ Treatment with VA for xerophthalmia and measles is part of the Integrated Management of Childhood Illness guidelines in South Sudan, but VAS for severely malnourished children is not recommended as ready to use therapeutic foods contain adequate VA, according to a key informant.

The MOH also recommended VAS for all women postpartum (200,000 IU) within 8 weeks of giving birth,^21^ although this is now stated as within 1 week of delivery in a new MOH Mother and Child Handbook. VAS is intended to be given to women in health facilities. Because 75%–88% of women deliver at home,^21^^,^^45^ there is no mechanism to reach those women with VA, although the Mother and Child Handbook urges women to visit a health facility soon after delivery. However, the World Food Program provides super cereal plus (containing micronutrients) to malnourished pregnant and lactating women screened through their supplementary feeding program in disadvantaged areas.

#### VAS Coverage Trends

In 2006, VAS coverage in the southern states of Sudan was 39.8% for children aged 6–59 months (in some states it was linked to immunization campaigns) and 17.5% for postpartum mothers. Receipt of VAS throughout all of Sudan (inclusive of the southern states which is now The Republic of South Sudan) was similar across child age cohorts (6–11 months and 12–59 months) although there is no age-wise report for the southern states only.^20^ Results from the 2010 SHHS II survey (which was conducted as South Sudan was struggling to rebuild after decades of war) showed a large decrease in national VAS coverage to only 4% among children aged 6–59 months (received in the last 6 months as reported by caregiver) with no VAS coverage data for postpartum mothers reported.^21^ The highest coverage was observed among children aged 12–23 months at 16%. The VAS coverage rate in urban areas was 1.79 times that of rural areas and inter-state variation was significant from 7.7% in Upper Nile to only 1.15% in Warrap. The poverty difference and influence of mother's education on receipt of VAS were also considerable with only 2.3% of children from poorest households receiving VAS compared to 7.6% from richest households and children of mothers with some secondary education (9.9%) had higher VAS coverage than mothers with no education (3.0%).^21^ In 2011, the MOH conducted a national cross-sectional household survey using lot quality assurance sampling to establish a monitoring system baseline for decision making at the national, state, and county levels. Results of the study showed that VAS at the national level had improved to 30% among children aged 6–59 months with only 28/75 counties surveyed reaching the 50% coverage target.^46^ However, a second national cross-sectional household survey using lot quality assurance sampling done in 2015 showed a significant decrease in VAS coverage to 4.8% (+1.6%) when compared with the 2011 lot quality assurance sampling survey.^47^

As South Sudan was struggling to rebuild after decades of war in 2010, national VAS coverage dropped to only 4% among children aged 6–59 months.

In South Sudan, NID campaigns have been the primary VAS delivery mechanism. The number of children receiving VAC by age group (6–11 months and 12–59 months) has been recorded on tally sheets. However, the coverage data particularly in the early years are not reliable and are indicative only given that the denominator (population of children aged 6–59 months) estimates lack precision, which is partially due to mass movement of the population. Campaigns were meant to be conducted twice yearly during April/May and 6 months later (November/December) but sometimes got delayed for political or environmental reasons. During some years (2015, 2019, and 2020 [different counties distributed VASD at different times in 2020]), only 1 VASD distribution round occurred. In all other years, a limited geographic distribution took place during at least 1 of the distribution rounds. Some states had low numbers of children receiving VAC (and consequently low coverage) due to inaccessibility in some counties or the entire state related mainly to insecurity in 2014, 2015, and 2016; to adverse events from vaccinations in 2017; to massive flooding throughout the country in 2019 that shifted the second NID round to the first quarter of 2020 and covered only 55 counties; and to COVID-19-related travel restrictions in April 2020 that postponed the round in some counties to November and then reached only 13 of the remaining 22 counties. The December 2014 NID VAS distribution round and the period from November 2017 to May 2019 had the best geographic coverage with the greatest number of the 79 counties distributing VAS (Table 2). No clear pattern in the number of children provided VAC at the national level over the 7-year period can be found except for a steady upward trend from May 2017 through May 2019.

**TABLE 2.** VAS Delivered to Children Aged 6–59 Months During NIDs, South Sudan^a^

NID Year	Round and Month Data Collected	Target Population	VAS 6–59 Months	VAS 6–11 Months	VAS 12–59 Months	Reported Coverage, %	No. Counties (N=79)
2014	I, April	1,402,839^b^	1,547,673	413,410	1,134,263	110.3	44
2014	II, December	1,968,213^b^	1,983,386^c^	520,255	1,333,187	100.8	77
2015	No round I conducted						
2015	II, November	1,999,080^b^	1,925,526	336,922	1,588,604	96.3	40
2016	I, April	3,032,720	1,394,243	330,701	1,063,542	46.0	34
2016	II, November	3,032,720	2,370,989	343,219	2,027,770	78.0	64
2017	I, May/June	3,032,720	1,536,612	310,913	1,225,699	50.9	48
2017	II, Nov/Dec	3,048,778	1,844,844	304,095	1,540,749	60.5	73
2018	I, April	3,032,720	2,269,615	423,748	1,845,867	74.8	72
2018	II, November	3,054,375	2,350,747	471,499	1,879,248	77.0	73
2019	I, May	2,965,795	2,766,172	596,694	2,169,478	93.0	78
2019	No round II conducted due to massive flooding in most parts of country						
2020	I, Feb/March and November^d^	3,002,984	1,867,524^c^	328,132	1,338,972	62.2	68

Abbreviations: NID, national immunization day; VAS, vitamin A supplementation.

a Data source: summary coverage reports of NID tally sheet data from 2014–2020.

b Target population is estimated only for those areas where VAS was delivered, not a national estimate.

c VAS recorded only for age 6–59 months category (not by ages 6–11 months and 12–59 months) in Western Bahr el Ghazal in 2014 and in Lakes in 2020.

d In 2020, there were no NIDs so VAS and deworming was piggybacked on the measles vaccination follow-up campaign, which reached 68 counties in 2 phases (55 counties in February/March and 13 counties in November).

An analysis of the frequency that each state achieved at least 80%, 70%, 60%, or lower VAS coverage over the 11 NID/VAS distribution rounds was done (Table 3). States with more rounds with low coverage are Jonglei, Unity, and Upper Nile, and the states with more rounds with high coverage are Lakes, Northern Bahr el Ghazal, and Western Bahr el Ghazal. In 2020, more than 80% coverage was reported in Northern Bahr el Ghazal (106%), Eastern Equatoria (90%), and Western Equatoria (84%), with the lowest coverage reported in Upper Nile (24%) and Unity (41%).

**TABLE 3.** Indicative VAS Coverage Trends From 11 NID Rounds Between 2014 and 2020, South Sudan

State	No. NID Rounds ≥ 80%	No. NID Rounds ≥ 70%	No. NID Rounds ≥ 60%	No. NID Rounds < 60%
Central Equatoria	6	8	9	2
Eastern Equatoria	5	7	7	4
Jonglei	0	3	4	7 (3 with no distribution)
Lakes	10	11	11	0
Northern Bahr el Ghazal	8	8	10	1 with no distribution
Unity	2	2	3	8 (4 with no distribution)
Upper Nile	0	0	1	10 (5 with no distribution)
Warrap	7	8	9	2
Western Bahr el Ghazal	8	8	9	2 (1 with no distribution)
Western Equatoria	6	8	9	2 with no distribution

Abbreviations: NID, national immunization day; VAS, vitamin A supplementation.

a Data source: summary coverage reports of NID tally sheet data from 2014–2020.

It is not possible to compare VAS coverage for women postpartum between 2006 and 2020 given the data were not collected during FSNMS surveys or in the government's health information system (District Health Information System 2); nor has treatment with VAS been specifically tracked in any database.

#### VASD Coverage

The FSNMS surveys collect data on children aged 6–59 months who received VASD in the last 6 months as reported by the caregiver or seen on the child's health card (which are seldom available). It includes VASD received through NIDs, as stand-alone VASD campaigns, during integrated rapid response missions, or routine immunization visits, and any follow-up of missed children door-to-door or at the health facility (for prophylactic and treatment).

**FIGURE 2:**
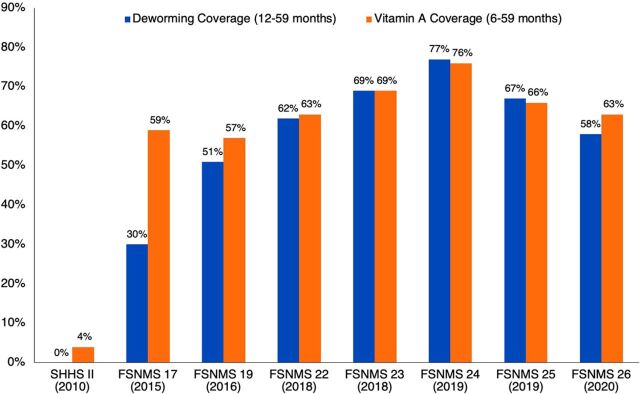
National VAS and Deworming Coverage of Young Children During Selected Survey Rounds, South Sudan, 2010–2020^a^ Abbreviations: FSNMS, Food Security and Nutrition Monitoring System; SHHS, South Sudan Household Health Survey; VAS, vitamin A supplementation. **^a^**Data source: SHHS II and FSNMS Survey Reports and FSNMS Round 26 raw VAS data.

At the national level, survey data showed that VAS coverage improved among children aged 6–59 months from 4.0% (2010, SHHS II) to 76.4% (August 2019, FSNMS Round 24). The increase was generally steady to the highest coverage point in August 2019 reflecting receipt of VAC through NIDs (in 78/79 counties of all states due to improved security and access) and via all other mechanisms (Figure 2). Coverage decreased to 66.0% in December 2019 (FSNMS Round 25) because there was no NID campaign in South Sudan during the 6 months before (June–November 2019) the survey. Respondents likely recalled receipt of VAC that happened 7 months before the survey during the May 2019 NID. Based on a preliminary analysis of the raw FSNMS Round 26 data, national level VAS coverage decreased to 63% for children aged 6–59 months (similar to the 2020 NID coverage of 62%) and deworming coverage was estimated at 58% among children aged 12–59 months in 2020. Deworming coverage generally mimicked VAS coverage in most years except in 2015 when it was much lower at 29.6% nationally, ranging from 4% in Warrap to 53.2% in Western Equatoria.

After VAS coverage increased to 76% in August 2019, survey data show it decreased to 66% in December 2019 because there was no NID campaign in the 6 months before the survey.

Given that there were several missed VAS distribution rounds and many subnational distribution rounds between 2015 and 2020, it is not useful to report trends in twice-yearly VAS coverage. Preliminary analysis of the FSNMS Round 26 data showed some differences in coverage by age with children aged 6 months and 7 months having the lowest coverage at 46% and 50%, respectively; and overall, children aged 6–11 months had lower coverage (56%) than children age 12–59 months (63%). Child cohorts at age 1 to 4 years had similar VAS coverage with age 1 at 64%, age 2 at 63%, age 3 at 64%, and age 4 at 62%. As noted above, the intra-country coverage variability across states (and within states) from survey round to survey round occurred for diverse reasons with some states (Western Bahr el Ghazal, Central, Eastern Equatoria, Western Equatoria, and Unity) closer to achieving 80% VAS coverage more frequently (Table 4). Conversely, Warrap, Jonglei, Northern Bahr el Ghazal, and Upper Nile had low coverage more often and have not achieved 80% coverage during any 6-month recall period (before the survey). Although Lakes has yet to achieve 80% coverage, it has improved toward a high of 76.2% in 2019 (FSNMS Round 24). Note that, except for Jonglei and Upper Nile (low coverage) and Western Bahr el Ghazal (high coverage), high and low coverage states in the FSNMS survey data are different than the NID data analysis for high and low performing states. This difference could be due to denominator issues in NID reports (as mentioned earlier) and mothers' subjective recall of VAS receipt during FSNMS surveys.

**TABLE 4.** Percentage of Children Aged 6–59 Months Who Receive VAC During the 6 Months Preceding Each Survey (VAS Coverage) by State and Nationally, South Sudan^a^

	Period of Data Collection (FSNMS Round)					
State	November/December 2015 (R17)	December 2016 (R19)	July/August 2018 (R22)	November 2018 (R23)	May 2019 (R24)	November 2019 (R25)
Central Equatoria	83	90	57	82	79	66
Eastern Equatoria	72	70	66	81	79	60
Jonglei	60	47	58	54	60	49
Lakes	58	59	70	72	76	70
Northern Bahr el Ghazal	61	52	61	63	76	55
Unity	--	62	69	83	83	70
Upper Nile	60	57	47	71	78	68
Warrap	40	44	50	56	76	49
Western Bahr el Ghazal	71	89	87	68	80	91
Western Equatoria	69	78	72	77	84	89
National	59^b^	57^b^	63	69	76	66^c^

Abbreviations: FSNMS, Food Security and Nutrition Monitoring System; VAS, vitamin A supplementation.

a Data source: FSNMS Rounds 17 to 25 Survey Reports that included VAS coverage statistics.

b Data based on weighted average from FSNMS reports.

c There was no VAS distribution in the 6 months before the survey.

**FIGURE 3:**
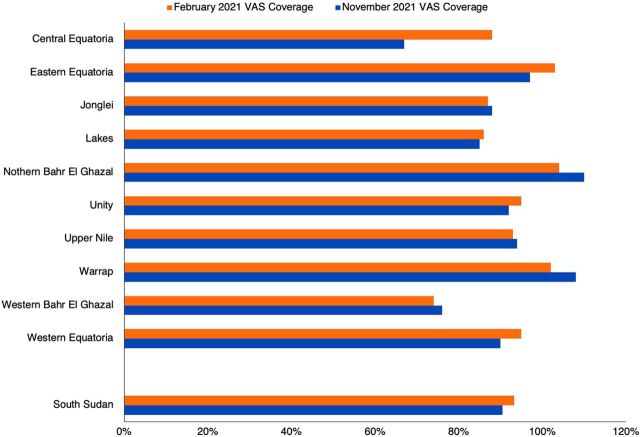
Children Aged 6–59 Months Who Received Vitamin A Capsules During Stand-alone Campaigns in February and November of 2021, South Sudan, by State and Nationally^a^ Abbreviation: VAS, vitamin A supplementation. ^a^ Data source: State/county coverage reports provided by the Ministry of Health and compiled by UNICEF South Sudan.

Two VASD stand-alone campaigns occurred in February and November 2021 throughout the country with VAC provided by the Government of Canada and albendazole provided by World Vision International. The campaign was implemented under the stewardship of the MOH (national, state, and county) with field support from development partners. Even though COVID-19-related restrictions were in place, with infection prevention control measures guided by Global Alliance for Vitamin A, all but 2 counties were able to distribute VASD during the February campaign, and all counties distributed VASD during November (although 3 counties were part of a pilot implementation described later). Figure 3 shows coverage for VAS was more than 90% at the national level (93% in February and 91% in November) and more than 80% in all states except Central Equatoria (67% in the February distribution round) and Western Bahr el Ghazal (74% and 76% in the February and November distribution rounds, respectively). Deworming coverage was generally a little lower than VAS coverage and at the national level was 86% in February and 88% in November among children aged 12–59 months. Central Equatoria had the lowest deworming coverage during November (65%) and Western Bahr el Ghazal had the lowest in February (62%). The summary tables (provided by UNICEF) showed VAS coverage for children aged 6–11 months of 178% and 183% in rounds 1 and 2, respectively, which implies issues with categorizing by age cohort, recording data, and/or incorrect denominators.

A stand-alone campaign in November 2021 helped distribute VASD to all counties, increasing VAS coverage rate to more than 90% nationally.

During the second semester of 2021 (November), the MOH and UNICEF initiated a pilot implementation in 3 counties (2 in Warrap and 1 in Upper Nile) to test delivery of VASD as part of quarterly screening for wasting, a community-based intervention. Preliminary results showed lower VAS coverage (48%) overall among children aged 6–59 months in routine versus campaign delivery (which was 108% in Warrap and 94% in Upper Nile). The VAS coverage among children aged 6–11 months was 100% in the routine distribution, primarily due to children receiving VAS at their 9-month measles vaccination contact at health centers. Deworming coverage in the routine pilot distribution was 49% among children aged 12–59 months, which is much lower than deworming via the campaign approach (which was 89% in Warrap and 90% in Upper Nile).

#### VAS With Other Indicators

FSNMS reports included associations of VAS with other indicators (although these could be in part a reflection of contact with health services):

- An FSNMS Round 17 analysis showed that states with the lowest measles vaccination and VAS coverage had the highest wasting rate. Although vaccination against measles, closely linked to mortality from VAD, was not optimal anywhere in 2015 or 2016 except in Central Equatoria in 2016, it tended to be highest in the high VAS coverage states.
- A bivariate analysis (FSNMS Round 19) showed a significant inverse correlation (P<.05) between wasting in young children and VAS. Children were more likely to have better nutritional status when they received VAS (sig .001, OR=1.443) and measles vaccination (sig .000, OR=1.627). There was a significant correlation between nutrition status of young children and VAS, deworming, measles vaccine, diarrhea, fever, overall morbidity, chronically ill person in the household, and access to health services.
- FSNMS Round 22 data indicated that Jonglei, with low VAS coverage, had the highest level of morbidity (fever, cough, and diarrhea) with 42.3% of children aged 6–59 months having 1 or more illness in the past 2 weeks. Round 23 data showed Central Equatoria, Eastern Equatoria, and Unity had the lowest morbidity rate at 61% each—the 3 states with the highest VAS coverage.

Other information sources may soon be available to track/verify VAS coverage:

- The District Health Information System 2 was revised (June 2020) to include 2 VASD indicators on the Child and Maternal Nutrition Monthly Summary Form (receipt of VAS and deworming at the fifth and ninth dose), but this has not been fully rolled out across the country and no data are yet available.
- The Mother and Child Handbook will record 9 doses of VAS for children aged 6–59 months. It is being piloted in selected health facilities with country-wide scale-up planned.
- The 2021 national VASD coverage data for South Sudan will be available for public access via Nutridash in July 2022 and a report documenting coverage and learning from the pilot to test integration of VAS into community-based screening will be available at the end of 2022.

### VASD in the Primary Health Care Sector

#### Challenges

Although progress has been made to reestablish health services across the country since the peace agreement was signed, the quality of and access to primary health care remains an issue. According to the FSNMS Round 21 (2017) findings, overall, 73% of respondents reported that community health facilities are open, but only 54% said the facilities had the capacity to deliver services. Data (as reported by respondents) also showed that the mean travel time to any health facility was 6 hours (and in some areas, it takes more than a day or requires flying to get to the nearest county/state referral hospital), with the average distance at 9.3 km (ranging from 1.3 km to 19.7 km).^26^ Insecurity and tribal skirmishes also cause fear to travel or participate in health-related activities in some areas. Although health services are supposed to be free, the quality of health care is affected by the lack of funding to support health workers. According to a key informant, because community health workers (CHWs) are not regularly paid, some lack the motivation to serve people causing community members to not have confidence in them. Lack of funding for transport also impedes outreach services by nurses and CHWs within their catchment areas. Other challenges to delivering health services include inadequate numbers of trained staff, poorly equipped health facilities, and periodic breaks in the supply chain (although this does not seem to be an issue for VAC as they are financed by Canada through UNICEF).

#### Preliminary Success

Some priority nutrition programs are working relatively well and reaching the marginalized areas with financial and organizational assistance from nongovernment partners and United Nations agencies. Since 2011, due to the high level of wasting among young children and women, the government of South Sudan has prioritized screening, referral, and treatment for wasting throughout the country every 3 months as a critical component of outreach through routine health services. The program screened 1,782,942 children (aged 6–59 months) and 1,087,646 pregnant and lactating women in 2020.^48^ Counseling of pregnant and lactating women on MIYCN through community nutrition volunteers (CNVs) and mother-to-mother support groups is also an effective priority program with attendance exceeding targets in most counties.^48^ These groups have been shown to be effective at increasing key MIYCN practices, such as early initiation of and exclusive breastfeeding among mothers.^49^

#### Future Strategies

Reviews conducted by the MOH and partners highlighted the importance of developing a cost-effective and more sustainable means to deliver VASD to all children, given that polio NIDs were being phased out. It was proposed that VASD could be integrated into quarterly screening for wasting since it is a relatively well-established and monitored program that is community owned, hence, more likely to be sustainable. A national guideline was developed to operationalize the recommended approach. The intention is to reach all eligible children with VASD every 6 months and conduct screening for wasting every 3 months at one time and place through routine service delivery outreach. As mentioned in the VASD coverage section, a pilot implementation in 3 of 79 counties is ongoing to test the approach with additional assessment being done to better understand the findings to improve the implementation model for gradual scale up to additional counties in November 2022.

Delivery of VAS directly through CNVs (with support of mother-to-mother support groups), to each child in their catchment area when the child reaches age 6 months and every 6 months thereafter until the child reaches 59 months could also be effective at reaching vulnerable children in a sustainable manner. In either model, linkage to the established health structure for planning, budgeting, and procurement will be vital to ensure an ongoing supply of VAC throughout the year to all CNVs (although in the pilot previously mentioned, the MOH preferred to handle routine VAS through health center contacts and only allowed referral by community networks as is the current policy).^48^ An effective supportive supervision, monitoring, and evaluation mechanism would also be needed for quality control.

Delivering VAS directly through CNVs and mother-to-mother support groups to children in their catchment areas at age 6 months and every 6 months thereafter until the child reaches age 59 months could be effective at reaching vulnerable children in a sustainable manner.

For the immediate future, due to restrictions imposed by the COVID-19 pandemic, extraordinary measures will be in place to reach all vulnerable children with VASD twice yearly. Currently, routine programs may not be as effective to distribute large quantities of VAC everywhere so VASD stand-alone campaigns (while continuing to pilot, adapt, and scale up VASD delivery with quarterly screening for wasting) will continue to be implemented through 2023 with financial support from development partners.

## DISCUSSION

Under very difficult circumstances, South Sudan's VAS program has demonstrated success showing considerable improvement between 2010 and 2019 from 4% to 76% at the national level when delivered via NID campaigns and over 90% coverage in 2021 through 2 stand-alone VASD campaigns.

Nevertheless, South Sudan presents a unique operational context that impacts VAS coverage with different challenges at different points in time from area to area. Low coverage may be due to shifting civil and political unrest, environmental factors (natural disasters, difficult terrain, and distance to health posts), and/or structural and organizational inefficiencies (in the health system). For example, in 2013, the 3 states of the Greater Upper Nile region were badly affected by war, resulting in only a few counties being accessible for VAS. In 2016, the 3 states of the greater Equatoria became largely inaccessible due to a war that broke out in Juba resulting in low VAS coverage in this region. That said, the South Sudan data clearly shows that there is an uneven intra-country coverage with some states (such as Warrap, Jonglei, and Northern Bahr el Ghazal) consistently having lower VAS coverage than the national average.^22^^,^^24^^,^^27^^–^^30^ But even within states and likely within counties, there are pockets of low or no coverage. Understanding the reasons for high and low coverage and prioritizing micro-planning at the state and county levels to tailor or augment the delivery approach will be important to achieve equity. Having a local supply of VAC (although storage of VAC in boma could be challenging) and trained staff to deliver it in areas that are frequently inaccessible would facilitate distribution every 6 months. On a broad level, assessing the intra-country coverage variability and targeting low-performing areas before ending VAS campaign-style supplementation programs everywhere, should also be considered.

South Sudan presents a unique operational context that impacts VAS coverage with different challenges at different points in time from area to area.

To add to this inequity, studies have shown that even well-performing VAS programs do not always reach the entire target population, particularly in remote areas.^50^ Approximately 10%–20% of those children missed by VAS may be those most in need of VA, as they have more morbidity from infections, come from poorer households, are more malnourished, have fewer contacts with the health system, come from families with higher infant and child mortality rates, and may be younger (6–11 months).^51^ Care must be taken to include those children and conduct systematic tracking and follow-up of any missed children in the delivery approach. Using the vast FSNMS data set, more analysis could be done to understand who is being missed, where they are located, and why they are being missed. GPS could be used to conduct mapping to ensure delivery of VASD to those children.

Additionally, in low-resource countries, it is important to reach children with VAS exactly at age 6 months as we know that newborns have very low hepatic VA reserves, and younger children have the highest risk of mortality from VAD. Although infants who are exclusively breastfed to age 6 months are generally protected against VAD,^52^ in South Sudan, the exclusive breastfeeding rate is 68.1%, discarding colostrum is common, few mothers receive VAS postpartum, and women's nutrition and VA intake are poor so their breastmilk retinol may be low. Increasing a mother's breastmilk retinol would have positive effects for both mother and child,^53^ thus additional promotional efforts should be made to improve that and all the above practices to better ensure adequate VA status among children at age 6 months. Regarding delivery of VAS, an analysis done in sub-Saharan Africa found that linking VAS to a 6-month visit reduced mortality by an additional 1.63% to 1.98%, compared to child health days (which reached only 16.7% of children at 6 months) or a 9-month health contact (like measles immunization), respectively.^54^ The recent community-based implementation pilot in South Sudan showed high coverage among children aged 6–11 months but mainly through the 9-month health contact. The FSNMS Round 26 preliminary analysis showed that when delivered via campaigns, VAS coverage was lowest among children aged 6 and 7 months.

With the delivery mechanism changing in South Sudan, it would be worthwhile to collect and report data for the index child by age in months over several FSNMS rounds to better capture coverage among the youngest children to understand who is being missed and why. In any case, reaching the youngest children with VAS could reduce U5M further; and integrating VAS into an effective community-based approach that targets children aged 6 months would accomplish this. Some countries, such as Nepal, have addressed the problem by implementing a hybrid approach with 6-monthly campaigns targeting all eligible children and trained female community health volunteers identifying and dosing the youngest children at age 6 months on an ongoing basis.^55^

In areas of the world with low U5M and low levels of infection (particularly measles and diarrhea), VAS may no longer be cost effective at reducing population-based young child mortality and morbidity.^11^ However, South Sudan still has high U5M and high infection rates at the national level and subnational areas that remain even more marginalized with poor access to the health system. Therefore, VAS will continue to have an important impact on saving young children's lives and reducing severe morbidity for the foreseeable future.

How can high VASD coverage be ensured among all preschool-aged children given that polio NIDs were phased out in 2020, funding for VASD stand-alone campaigns is only available through 2023, and the health system is not fully functional? A community outreach approach, integrated and firmly linked to the closest health facility, would likely achieve good results if CHWs and CNVs are well trained and provided with adequate supplies and if strong social mobilization efforts are made to ensure the most vulnerable families participate in routine services and outreach activities. The experiences of other sub-Saharan African countries (Ethiopia and Senegal, for example) transitioning from campaigns to delivering VAS through routine health services indicate that coverage initially drops significantly (particularly for children aged 12–59 months). However, over time, with good communication, training, supportive supervision, and adequate decentralized management and funding, coverage can improve.^56^

Conducting child health events once or twice yearly in the entire country or the most disadvantaged parts has resulted in high VAS coverage in many countries (such as the Philippines) and has the advantages of delivering multiple services with funding and coordination of multiple donors and government ministries.^57^ These events can serve to strengthen the health structure given that to be effective, the health system must be engaged at all levels for planning, budgeting, and logistics support. A hybrid approach is now being used in several countries such as Nepal and Tanzania, where VAS is delivered through campaigns in low-performing districts only and through routine health services everywhere, particularly to reach the youngest children. A strengthened VA supply system and extra support to low-performing districts have contributed to increased VAS coverage among all children. A hybrid approach to delivery may also be optimal for most areas of South Sudan but particularly in sparsely populated or remote areas. This approach could include 6-monthly campaigns for all children aged 6–59 months, community- and facility-based distribution especially for children as they reach age 6 months, and community-based follow-up door-to-door to reach the missed children. Policy change that ensures CNVs have a small constant supply of VAC for the children aged 6 months and that permits them to deliver VAC independently in villages will be critical. All of these event-type and community-delivery mechanisms should be tested in the different ecological, demographic, and political areas to see what works and what does not work there and to adapt for eventual scale-up as an individual or hybrid strategy. Recording, reporting, and close monitoring must be integral to any program to maximize VAS coverage by ensuring timely adjustments to distribution modalities.

A hybrid approach to delivery may also be optimal for most areas of South Sudan but particularly in sparsely populated or remote areas.

Many factors contribute to the persistent VAD problem in South Sudan, and therefore, designing, funding, and implementing a multisectoral, comprehensive strategy is necessary to address and eventually reduce VAD so it no longer constitutes a public health problem there. These interventions would benefit all vulnerable populations with high nutrient requirements (i.e., infants, preschool and school-aged children, adolescents, and pregnant and lactating women). These VA-specific and VA-sensitive interventions should encompass promotion of colostrum, exclusive breastfeeding from birth to age 6 months, prolonged breastfeeding to age 2 years and optimal complementary feeding with VA-rich foods from age 6 months to 2 years, dietary diversification with improved production and consumption of VA-rich foods, and fortification of staple foods with VA, coupled with control of infectious disease particularly measles (by improving immunization rates and ensuring timely treatment) and diarrheal disease (by improving access to and use of water, sanitation, and hygiene and ensuring timely treatment). Prioritizing nutrition and allocating resources at all levels of the health structure will be critical to ensure adequately trained staff, ample supplies, well-equipped health facilities, necessary promotional activities, and transport and strategic planning are available over the long term.

Unfortunately, there are still considerable obstacles to addressing the underlying problems of VAD in South Sudan. The unstable social and political situation continues to exacerbate food insecurity and malnutrition. In most areas of the country, families are unable to produce adequate food themselves, buying much of their food and thus, spending up to 80% of their very limited household income on food.^30^ Although variable by ecological zone, much of the South Sudan population subsists on mainly grains (primarily sorghum and maize) with very few animal foods (containing retinol) or plant food (rich in beta-carotene) consumed.^58^ And even if these foods are available in households, women and young children do not often eat adequate amounts to meet their nutritional and VA needs, in part due to the patriarchal nature of the society in most areas where women have little power in the household.^44^ To compound this problem, as data from the FSNMS shows, children (and people in general) in South Sudan continue to have frequent infections largely due to suboptimal access to and uptake of water, sanitation, and hygiene supplies and practices and below optimal deworming coverage, thusly depleting the stored VA that they do have in their bodies. Additionally, although South Sudan has comprehensive health and nutrition policies and strategies, due to limited resources, implementation is sporadic and highly dependent on external sources.

Lessons learned from other countries could provide a path to reduce VAD in South Sudan to low levels permanently. Many countries, such as Bangladesh, Cambodia, Nepal, and Burkina Faso, have successfully implemented dietary diversification approaches that have increased the production and consumption of VA-rich foods, improved household food security, and improved women's agency, but scaling them up has been problematic.^55^^,^^59^ Fortification of commercial products (such as oil, wheat flour, and sugar) have also been shown to be effective at sustaining adequate serum retinol levels.^60^ This strategy has been successfully implemented in some LMICs but is currently only at the discussion stage in South Sudan. It requires a central processing system, an effective quality control mechanism (not yet functional in South Sudan), a wide distribution network to ensure the most marginalized areas are reached, affordability by the poorest households, and nutrition education to optimize usage. Biofortification with beta-carotene of some crops (such as sweet potato, sorghum, cassava, maize, and rice) is already done or underway and, if introduced into the country, could help contribute to improved intake of VA among vulnerable groups and reduce VAD.^61^ Conversely, as South Sudan takes steps to transition from an emergency health system to sustainable health system development, the process of transitioning from vertical programming for VASD to sustainable community-centered programming could provide lessons for South Sudan as well as other fragile nations emerging from conflict. The use of local data to guide decisions on what type of intervention(s) to implement in the shorter- and longer-term should also be documented carefully so that other countries could follow a similar process (if it works).

As South Sudan transitions from an emergency health system to sustainable health system development, the process of transitioning from vertical programming for VASD to sustainable community-centered programming could provide important lessons.

## CONCLUSION

South Sudan's VAS program has demonstrated success through campaign-style delivery with steady improvement from 2010 to 2019 and high coverage during 2021. Given the country likely has a severe public health VAD problem and its underlying determinants are not likely to improve soon, VAS for young children will need to remain a key lifesaving and morbidity-reducing intervention until other interventions are in place to address the underlying problems.

A hybrid approach with both campaign and community-based distribution could best ensure the most vulnerable children—the youngest and those from marginalized households—are reached to further reduce the U5MR. In the short to medium term, to maintain high VAS coverage, South Sudan should continue twice yearly VASD campaigns with extra efforts made to improve coverage in low-performing areas, to follow up missed children, and to reach children at age 6 months. A multisectoral strategy, such as child health events, should be considered, at least in the most disadvantaged areas of the country. For the longer term, integrating VASD into a community-based and community-led intervention, such as quarterly screening for wasting, should be prioritized since it is a well-established government priority program with a felt need among the population. Mother-to-mother support groups and CNVs could also be effective community-driven delivery and promotional avenues for VASD and could encourage consumption of VA-rich foods among women and children.

Once tested distribution models are available, data from the FSNMS and District Health Information System 2, among other sources, should be used to guide decision makers when to adjust implementation strategies to ensure high-risk VAD populations receive timely VAS.

## Notes

**Peer Reviewed**

**First published online:** June 7, 2022.
